# Dietary Fiber: An Opportunity for a Global Control of Hyperlipidemia

**DOI:** 10.1155/2021/5542342

**Published:** 2021-04-08

**Authors:** Ying Nie, Feijun Luo

**Affiliations:** ^1^School of Food Technology and Biological Science, Hanshan Normal University, Chaozhou 521041, China; ^2^Laboratory of Molecular Nutrition, College of Food science and Engineering, National Engineering Laboratory for Deep Processing of Rice and Byproducts, Central South University of Forestry and Technology, Changsha 410004, China

## Abstract

Dietary fiber has a long history in the intervention study of hyperlipidemia. In this review, current understandings of structures, sources, and natures of various kinds of dietary fibers (DFs) were analyzed first. Available evidences for the use of different varieties of DFs in the lipid-lowering action both *in vitro* and *in vivo* were subsequently classified, including both soluble ones, such as glucans, pectins, and gums, and insoluble ones, including arabinooxylans and chitosans, in order to draw a primary conclusion of their dose and molecular weight relationship with lipid-lowering effect. Their potential mechanisms, especially the related molecular mechanism of protective action in the treatment and prevention of hyperlipidemia, were summarized at last. Five major mechanisms are believed to be responsible for the antihyperlipidemic benefits of DFs, including low levels of energy, bulking effect, viscosity, binding capacity, and fermentation thus ameliorating the symptoms of hyperlipidemia. From the molecular level, DFs could possibly affect the activities of HMG-CoA reductase, LDL receptors, CYP7A1, and MAPK signaling pathway as well as other lipid metabolism-related target genes. In summary, dietary fibers could be used as alternative supplements to exert certain lipid-lowering effects on humans. However, more clinical evidence is needed to strengthen this proposal and its fully underlying mechanism still requires more investigation.

## 1. Introduction

Cardiovascular disease (CVD), including atherosclerosis, stroke, and myocardial infarction, is a leading factor to cause death in modern industrialized societies, which indicates that to reduce the risk factors for CVD plays a critical role in the management of public health, including decreased concentrations of plasma total cholesterol (TC), low-density lipoprotein cholesterol (LDL-C), and triacylglycerols (TG). It has been estimated that a reduction in LDL-C for 10 mmol/L correlated with a 22% reduction in the risk of CVD mortality and morbidity and greater than 10 mmol/L TG concentrations is associated with significantly increased risk of acute pancreatitis and CVD [1, 2]. Besides these factors, high levels of plasma high-density lipoprotein cholesterol (HDL-C) are also found to be inversely related to the risk of CVD, although the application of state-of-the-art medical interventions targeting on HDL-C to reduce the CVD burden failed to show the anticipated beneficial effect.

Cholesterol, a subtype of lipids, originates from two sources in the human body; one is synthesized in the liver (about 700-900 mg/d), and another is taken in by diet (about 300-500 mg/d). In spite of small amounts of cholesterol that are used for the synthesis of steroid hormones and cell membranes, most of them are used by the liver to synthesize bile acids, such as cholic acid, which is essential to increase the absorption of hydrophobic nutrients. Bile acids are stored in the gall bladder and released into the duodenum and proximal jejunum after the stimulation of cholecystokinin. About 95% of excreted bile acids are reabsorbed and recycled by reuptake through enterohepatic circulation, and the lost fraction is compensated by synthesis and diet uptake. Cholesterol is critical in the lipid metabolism for it participates in the transportation of lipids as the form of lipoproteins, which are small spheres containing phospholipids, apolipoproteins, and cholesterol. These lipoproteins can be divided into subgroups: LDL-C, HDL-C, and VLDL-C. LDL-C delivers fat molecules to the cells and can drive the progression of atherosclerosis if they become oxidized and form plaque within the walls of arteries. Conversely, HDL-C is considered “good” cholesterol that protects against cardiovascular diseases and helps scavenge LDL-C from the arteries and carries it back to the liver, where it is broken down [3, 4]. Given the central role of cholesterol in the lipid metabolism, a reduction of cholesterol is closely related with a lowering action of other lipids. Understanding the process of lipid metabolism will help to reveal the underlying mechanisms of lipid-lowering action and its molecular mechanism.

Hyperlipidemia, referring to a metabolism disorder, is a common disease in modern society along with an unhealthy diet and less physical activity and has been regarded as the most crucial risk factor leading to CVD [5]. Its diagnosis is often based on the abnormal deviation of one or several plasma lipids, which composed of increased TG, TC, and LDL-C and decreased HDL-C. Measures to adjust the dysfunction of lipid metabolism is commonly agreed to prevent the outbreak of CVD, including restricted diet of less carbohydrates and fats and more exercise plus medicine intervention. However, hyperlipidemic patients regularly taking drugs, such as statins and fibrates, have various complaints about their adverse effects or contraindications [6, 7]. Meanwhile, a number of functional foods or nutraceuticals, such as plant stanols and sterols, soya protein, garlic, *β*-glucans, and other dietary fibers, have also been identified with antihyperlipidemic function [8, 9]. Dietary fiber, “the seventh nutrient” of the body, is particular and nonreplaceable to maintain the health and composed of two types: hydrosoluble and insoluble ones, and they are classically believed to exert their influence on lipid metabolism in different patterns: hydrosoluble fiber forms an unstirred water layer on the intestinal wall and delays the absorption of sugar and fat, while the insoluble ones increase the volume of the stool. However, this categorization is criticized for its inaccuracy to represent the effects of all fibers in the lipid-lowering action [10].

The impactions of dietary fiber on reducing serum lipids stem from a study carried out in the 1970s. At that time, the same dose between 2 and 10 g/d of oats, pectins, or guar gum was regarded as equal in the action of reducing TC and LDL-C [11]. Recent analyses have focused on specific fiber type, especially the molecular weight, indicating that different kinds of dietary fibers may not be equal, and there is a dose-response relationship that existed between fiber intake and the effects of lipid reduction. In the present paper, we summarized the relationship of different structures, molecular weights, and doses of each kind of dietary fiber and their antihyperlipidemic effect and their underlying mechanisms.

## 2. Structures, Compositions, and Sources of Different DFs

Dietary fiber has a long history of multiple health beneficial effects, preventing or withstanding a series of common diseases, including cancer, diabetes, hyperlipemia, CVD, series of intestinal diseases, and obesity [12, 13]. The major subtypes of DFs consist of cellulose, hemicellulose, chitosan, pectin, *β*-glucan, gum, and pectin, which all refuse to be hydrolyzed by any digestive enzymes secreted by nonruminant animals. Therefore, the majority of DFs cannot be absorbed by the small intestine and is thus utilized by the intestinal microorganisms for fermentation in the caecum and colon. Though plants usually harbor both hydrosoluble and insoluble DFs, their ratio differs according to the species and maturity degree of the plant.

Cellulose, hemicellulose, lignin, and chitosan compose the whole part of insoluble DFs. Binding as many as 10,000 D-glucose residues with *β*-l,4-glycocisidic and *β*-l,6-glycocisidic bonds forms a tree-like molecule, defined as cellulose, which is the base of the microfibril structure and the reason why it absolutely cannot be dissolved in water. Hemicelluloses are assembled with a series of heterogenic monosaccharides, and most of them are also insoluble in water but in alkali solutions. Various cereals contain hemicellulose, which consists of mainly arabinoxylans and arabinogalactans with mainly xylans or galactans as backbone and arabinose or pentosans as side chains. The water solubility of hemicellulose is strongly affected by the branching and substitution: the more branched the molecule is and the more hydrophilic the substitutions are, the more soluble the hemicellulose will be [14]. Lignin constitutes with amorphous molecules of phenyl propyl alcohol or its derivatives and exists widely in plant xylem, which is seldom considered edible. This paper abandons the discussion of lignin for this reason. Chitosan is derived from chitin with alkaline deacetylation, which composes of *β*-1-4-linked D-glucosamine and N-acetyl-D-glucosamine, and participates in the forming of the exoskeleton of crustaceans and the cell walls of fungi. Chitosan can exist either in a primary and unorganized structure or in a microcrystalline form, which normally can be hardly resolved in water.

**Figure 1:**
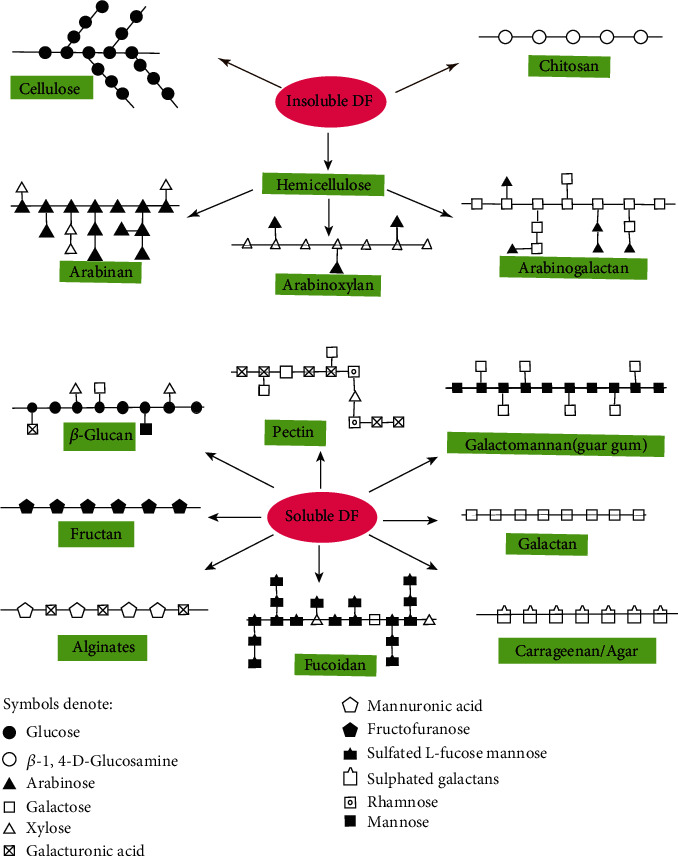
Structures and classifications of typical dietary fibers.

Most other DFs are water soluble, including *β*-glucan, alginates, carrageenans, agar, pectin, gum, and some prebiotics. The endospermic cell wall polysaccharides of wheat, oat, and rye are mainly water soluble, whose major constituents are *β*-l,3- and *β*-l,4-linked glucose and the *β*-l,3-linkages usually appear after several *β*-l,4-linkages [15]. Glucan is also commonly stored in various kinds of fungus, such as *Ganoderma* and *Lentinula edodes*, and thus attaching them with biological activity. The fungi glucans are characteristic of *β*-1,3- and *β*-1,6-linkages while *β*-1,3- and *β*-1,4-linkages appear in cereal glucans. The backbones of these glucans are composed of mixed *α*- and *β*-D-glucan or pure *β*-D-glucan while their side chains are heterogeneous of xylose, mannose, galactose, or uronic acid. Based on the different components and structures, these glucans can be both water soluble or insoluble [16]. The insoluble parts of fungi glucans, holding about 50–80% of the total glucan, form the structural components of the cell wall and are usually cross-linked to other molecules like chitin or to proteins [17]. Seaweed includes red, brown, green, and blue ones, and their polysaccharide extracts vary from one another, which can be further divided into glucans, alginates, carrageenans, agar, and focoidan for their different compositions. The structure of seaweed glucans showed by NMR spectroscopy is composed of two parts: *β*-1,3-glucan as backbone and about another one-fifth of *β*-1,6-glucan as sidechains [18]. Alginates are typically bonded with hundreds of D-mannuronic acid and L-guluronic acid residues. Carrageenan and agar are sulphated galactans with basic linear structure of galactose residues, but they differ from each other in whether the 1,4-linked anhydrogalactose is D or L form [19, 20]. Fucoidans are acidic and sulfated macromolecules composed of L-fucose along with several other oligosaccharides such as mannose, galactose, and xylose, which are usually extracted from brown algae like *Fucus vesisulosus* and sporophyll of *Undaria pinnatifida*. Pectin can dissolve in hot water and form a gel when it cools down, which owns an extremely diverse structure, but the main monomeric residue remains almost unchanged, mainly D-galacturonic acid with interruptions of rhamnose or galactose. Three major kinds of pectin molecules are recognized: homogalacturonan, rhamnogalacturonan-I, and rhamnogalacturonan-II [21], and they exist not separately but to form covalently linked domains. Plant-secreted gums usually possess highly branched structures and thus are highly water soluble. The molecules of endosperm cell walls of leguminous seeds are galactomannans, which are usually referred to as guar gum or locust bean gum. Fructooligosaccharides (FOS) and fructans are widely stored in various plants like garlic, onion, leek, chicory, and green algae. They vary in both molecular structure and weight: FOS are a common name for only lower polymers, referring to less than 10 fructooligomers, while fructans are molecules with a high degree of polymerization. Fructans may be classified into three main types: inulin, levan, and the branched ones. The inulin consists of fructose that connected mostly or exclusively with 1,2-linkages while levan fructose residues are joint mostly or exclusively with 2,6-linkages, and the branched ones contains both [22]. FOS and inulin are often served as prebiotics in modern functional food industries. The structures of the most typical DFs are illustrated in Figure 1.

## 3. Lipid-Lowering Effect of Different DFs

Based on current knowledge, certain kinds of nutraceuticals could exert significant lipid-lowering activity, although not effective enough to compare with statins, such as plant sterols and stanols, red yeast rice extract, garlic, bergamot, green tea extracts, and multiple kinds of soluble dietary fiber [23].

### 3.1. Oat or Oat *β*-Glucans

*β*-Glucan is an important dietary fiber with a biological function, which existed mainly in yeast, bacteria, oats, and barley as well as medicinal mushrooms. There are hundreds of studies involved in the effect of oat *β*-glucan on metabolism diseases. A meta-analysis that included 17 RCTs (916 hypercholesterolemic patients) showed that *β*-glucan consumption significantly reduced LDL-C (-0.21 mmol/L (8.1 mg/dL); 95% CI, -0.27 to -0.14; *p* < 0.00001), [24]. In a randomized, single-blind, wheat bran–controlled study, it suggested that consumption of 11 g oat bran *β*-glucan nearly doubled the plasma secretion of bile acids within 8 h and thus decreases serum cholesterol by measuring the metabolite 7-hydroxy-4-cholesten-3-one in the plasma [25]. Similarly, the supplementation of oat *β*-glucan (5 g/meal, twice a day) muesli diet significantly increased the synthesis of bile acid and lowered cholesterol absorption when compared to the control diet. It further suggested that the combination 5 g oat *β*-glucan plus 1.5 g plant stanols per meal enhances the lipid-lowering effect by reducing the absorption of cholesterol (*p* < 0.001) while the synthesis of bile acid remains unchanged [26]. Another study found that young adults consumed 6 g *β*-glucan containing oat bran diet daily for 2 weeks and had significantly lowered their TC, TG, LDL-C, VLDL-C, plasminogen activator inhibitor-1 (PAI-1), and factor VII (fVII), as well as fecal volumes and dry matter of the experiment group than the control group, while both groups had no significant differences in body weight [27]. Multiple studies verified that high level of PAI-1 is correlated with increased risk of CVD while both PAI-1 and fVII could influence postprandial TG levels [28, 29]. Another meta-analysis that included 28 randomized controlled trials declared that oat *β*-glucan in doses of 3 g/d reduced LDL-C and TC relative to control by 0.25 mmol/L and 0.30 mmol/L, respectively, without changing HDL-C or TG [30]. A recent 6-week randomized controlled trial was designed to assign 87 mildly hypercholesterolemia patients to one of the three groups: control or low dose of oat *β*-glucan (1.5 g/d, OL) or high dose (3.0 g/d, OH), whose plasma TC levels are situated between 5 and 7.5 mmol/L. Results showed that while TC reduced significantly in all groups, only OL and OH reduced significantly the plasma LDL-C and the intake of 1.5 g/d was proved to be as effective as the dose of 3 g/d irrelevant with different food formats [31]. However, another clinical trial aiming to test the effects of physicochemical properties of *β*-glucan on its ability to lower serum LDL-C found that the *β*-glucan must be served with sufficient quantity (3 g/d) and the efficacy of oat *β*-glucan in lowering blood TC was decreased by 50% when its molecular weight (MW) was reduced from 2,210,000 g/mol to 210,000 g/mol, suggesting that molecular weight plays an important role in the lipid-lowering action of oat *β*-glucan. Given that the viscosity of *β*-glucan is determined by its solubility and MW [32], this indicates further that mechanism of the lipid-lowering action of soluble *β*-glucan may be modulated by its physicochemical properties in the intestine. Similar results were found by another randomized clinical trial, in which 345 patients of both Caucasians and non-Caucasians were randomly assigned to consume cereal containing wheat fiber (control, *n* = 74 : 13 Caucasian : non-Caucasian) or 4 different oat *β*-glucan groups: 3 g/d of high-MW 2,250,000 g/mol (*n* = 67 : 19), 4 g/d medium-MW 850,000 g/mol (*n* = 50 : 17), 3 g/d medium-MW 530,000 g/mol (*n* = 54 : 9), or 4 g/d low-MW 210,000 g/mol (*n* = 51 : 12) for 4 weeks. Individuals that consumed medium to high MW *β*-glucan all had significantly reduced LDL-C by 4.8 to 6.5% in both race, but low-MW had no effect compared to control [33]. Moreover, a latest systematic review that included 58 trials also claimed that a median dose of 3.5 g/d of oat *β*-glucan significantly lowered LDL-C by 0.14-0.23 mmol/L (*p* < 0.00001), non-HDL-C by 0.15−0.26 mmol/L (*p* < 0.00001), and apoB by 0.02−0.05 g/L (*p* < 0.0001) compared with control [34]. However, there is also controversial result. 66 overweight females were randomized into one of three 2 MJ energy-deficit diets: a control and two interventions including 5–6 g or 8–9 g *β*-glucan. After 3 months, all groups lost weight (*p* < 0.001) and significant reductions in TC, LDL-C, HDL-C, and leptin while no significant differences were noted between the groups, suggesting that oat *β*-glucan cannot enhance the antihyperlipidemic effect in energy-restricted diets [35]. From the above evidences, it is commonly agreed that oat and its enriched *β*-glucans are effective lipid-lowering agents, 3 g/d may be the effective dosage, and low molecular weight *β*-glucans (less than 200 kDa) have minor hypolipidemic effect. However, what is the ideal MW of the *β*-glucans for hyperlipidemic patients needs further investigation.

### 3.2. Barley or Barley *β*-Glucan

Many studies pointed out that barley *β*-glucan also possesses lipid-lowering properties. Eight eligible trials lasted 4 to 12 weeks involving 391 subjects, which are aimed at evaluating the lipid-reducing effect of barley, were identified in a meta-analysis. It found that the intake of 3–10 g barley *β*-glucan lowered TC by about 14 mg/dL, LDL-C by about 10 mg/dL, and TG by about 12 mg/dL but did not significantly alter the HDL-C level [36]. A later meta-analysis conducted with 11 studies reached similar conclusions; supplementation of comparable barley glucan could lower TC and LDL-C concentrations by 0.30 mmol/L and 0.27 mmol/L, respectively; and this lipid-lowering action had no dose-dependent relationship [37]. Hamsters were fed with high-fat diets plus different kinds of grain including whole grain wheat, barley, barley supplemented with HPMC (2%-3%), debranned oat, and oat supplemented with HPMC, which were all compared to a diet containing cellulose as control. Results showed that all supplementations significantly lowered plasma LDL-C concentrations compared to the control and HPMC further strengthened the lipid-lowering effect both in the plasma and liver. It appears that whole grain barley especially when HPMC is applied could reduce the cholesterol mainly through modulation of the synthesis and excretion of hepatic cholesterol and bile acid [38]. Another study also supports the idea that consumption of 3 g/d high MW *β*-glucan from barley lowered TC effectively by circa 0.12 mol/L, while low MW *β*-glucan did not alter serum TC levels and even raise the dose to 5 g/d. This effect was further found to be correlated with gene-diet interaction, whereby individuals with G allele carriers of the CYP7A1 gene, namely, GG homozygotes or GT heterozygotes, exhibited more pronounced cholesterol-lowering effects than TT carriers (*p* = 0.0006) [39].

### 3.3. Mushroom Polysaccharides

Mushroom polysaccharides existed in varied forms including *β*-glucan. A great variety of active polysaccharide molecules, including heteroglucans, heterogalactans, and heteromannans have been obtained from various kinds of mushrooms such as fungi, basidiomycetes, and ascomycetes, whose biological function has been explored by abundant studies over the past decades [40]. In a study, *Pleurotus ostreatus* DF fraction (PDF) led to a reduction of hepatic TG because Dgat1 was downregulated in HFD mouse models. It could also reduce hepatic TG accumulation by modulating cholesterol-related gene expression in a manner similar to that of typical antihypercholesterolemic drugs including simvastatin and ezetimibe, although no significant change in plasma and liver biochemical data were noticed [41]. They further prepared 4 different mushroom extracts including *β*-glucans, water-soluble polysaccharides, ergosterol, and their mixture to examine the underlying molecular mechanisms involved in cholesterol-lowering action, in which the mRNA levels of 17 cholesterol-related genes from the jejunum, caecum, and liver of high cholesterol-fed mice were evaluated. The 4 tested supplements decreased plasma TC by 22−42% and LDL-C by 27−51%, and two of them increased mRNA levels of jejunal Npc1l1 and Abcg5 and hepatic Npc1l1, which indicates that the mushroom extracts could decrease dietary cholesterol absorption and increase bile acid excretion [42]. Zou et al. [43] developed a two-stage pH control strategy to enhance the production of polysaccharide in mushroom fermentation. Results showed that this mycelia zinc polysaccharide of 3.64 × 10^4^ Da improves both the blood and the liver lipid levels and the antioxidant status and attenuates the liver cell injury in hyperlipidemic mice [44]. These findings suggested that mushroom extracts including *β*-glucan and other water-soluble heteropolysaccharides may have potential to serve as the novel cholesterol-lowering functional foods.

Besides, *β*-glucan (source unknown) appeared to be more effective in lowering plasma LDL-C, TC, apoA-I, and glucose levels, compared with rice bran-enriched food in a 14-week trial [45]. Another meta-analysis that included 17 randomized controlled trials with 916 subjects showed that 3-10.3 g/d *β*-glucan consumption in hypercholesterolemic population significantly lowered TC by average 0.26 mmol/L and LDL-C concentration by average 0.21 mmol/L, with no significant differences in HDL-C, TG, and glucose, and no reports of adverse effects were received [24], which suggested the feasibility of *β*-glucan as adjuvant agents of antihyperlipidemia. According to the scientific opinion of EFSA, 3 g/d *β*-glucans from cereals including oats and barley or from mixtures of nonprocessed or minimally processed whole grain should be equal and served in one or more times to achieve the experimental hypolipidemic effect. However, some study criticized that a typical serving of cereals containing this amount of *β*-glucans requires more than 100 g/d [46]. In practice, this is quite difficult to realize unless this amount is separated into more than two portions per day. Maybe developing purified *β*-glucan products could help to solve this difficulty of application.

### 3.4. Konjac Glucomannan

12 male baboons were included in a 9 wk crossover, randomized trial, in which they were fed a typical western human diet with or without supplements of 5% konjac glucomannan (KGM). Serum TC levels were observed to be about 25% higher than baseline when baboons consumed the western diet without supplements while KGM could reverse this increase. KGM supplementation also led to significant reduction of TG from baseline values and circulating FFAs. Liver cholesterol concentration was 31-34% lower with KGM than with the western diet [47]. The effectiveness of 3.9 g/d KGM on a reduced serum cholesterol (10%, *p* < 0.0001), LDL-C (7.2%, *p* < 0.007), and TG (23%, *p* < 0.03) in men was also observed in a double-blind crossover, placebo-controlled 4 wk study back in 1995. Besides, this hypolipidemic effect of KGM is observed without adverse effects, showing that KGM is an effective cholesterol-lowering dietary adjunct [48]. A meta-analysis that involved 12 studies (*n* = 370), 8 in adults and 4 in children, declared that the intake of 3 g/d KGM significantly lowered LDL-C for 10% (MD: 20.35 mmol/L; 95% CI: 20.46, 20.25 mmol/L) and non-HDL-C for 7% (MD: 20.32 mmol/L; 95% CI: 20.46, 20.19 mmol/L) and 6 of them suggested no impact of KGM on apolipoprotein B [49] Another meta-analysis included 14 RCTs with 531 patients concluded that the use of glucomannan (dose ranging between 1.24 and 15.1 g/d) significantly reduces TC (WMD: -19.28 mg/dL; 95% CI: -24.30, -14.26), LDL cholesterol (WMD: -15.99 mg/dL; 95% CI: -21.31, -10.67), and triglycerides (WMD: -11.08 mg/dL; 95% CI: -22.07, -0.09) [50].

### 3.5. Pectins

Another viscous DF called pectin, distributed widely in cell walls of fruits and vegetables, consists of linear chains of *α*-1-4-galacturonic acid units with side chains including galacturonic and glucuronic acids and also shows a prominent blood cholesterol-lowering effect. By reference to an early meta-analysis, 7 relevant studies (*n* = 277 subjects) around the 1990s showed that the intake of pectins at 4.7 g/d caused a significant lowering effect on TC and LDL-C and there existed a significant dose-dependent relationship between the intake and the lowering effect, but no significant dose-response exhibited for HDL-C and TG [11]. A later study found that when hamsters were fed with high-cholesterol (0.1% *w*/*w*) diets plus 3% of lemon pectin or the same dose of the polygalacturonic acid region fraction of the lemon pectin for 8 weeks, both groups showed significant lower blood TC levels than the cellulose group. However, only the polygalacturonic acid region fraction group reached statistical significance in the lowering experiment of liver cholesterol which may suggest that the polygalacturonic acid regions of the pectin are responsible for the cholesterol-lowering action of the pectins [51]. The team studied further to find out whether the cholesterol-lowering effect of the peels of lemon contributed mainly from the pectin component using the same experiment model. Results suggested that lemon peel is as effective as the pectin extracted from the peels in lowering blood and liver cholesterol in hamsters [52]. Another study found the cooperation of pea proteins and apple pectin is extremely effective to reduce plasma cholesterol in rats by upregulating CYP7A1 and NTCP genes, which are involved in hepatic cholesterol turnover [53]. A 12-week, placebo-controlled, randomized, parallel double-blinded study enrolled 66 middle-aged patients with abnormal glucose metabolism to examine the effects of sugar beet pectin (SBP) or polydextrose (PDX) on fasting glucose and lipid levels. Both the SBP and PDX had an increase in fasting serum HDL-C concentration compared to the control [54]. Another study compared serum cholesterol-lowering effect of different nutritional supplements, including 30 g/d of pectin, 20 g/d of polyphenols, 6 g/d of phytosterols, and all possible combinations compared to 3 mg/kg of lovastatin using familial hypercholesterolemic (FH) swine. Although the effect of pectin is not the best during the 4-week experiment, however, both phytosterol and polyphenol enhanced the reduction in LDL-C of pectin. All supplementation group showed about a half of the efficiency of lovastatin to reduce TC in FH swine, which suggested the possibility of these diets alone or in combination with drugs to reduce LDL-C [55]. From the above evidences, it is not hard to recognize that pectin and other functional foods may serve as an adjuvant therapy agent for hyperlipidemia and it was suggested by the EFSA that in order to achieve the cholesterol-lowering effect on adults, it should provide more than 6 g pectins/d in one or more servings [56].

### 3.6. Brown Alga Polysaccharides

Alginates and fucoidan are usually extracted from brown algae like *Fucus vesisulosus* and sporophyll *of Undaria pinnatifida*. Multiple studies found that brown alga extracts also possess the hypolipidemic activity. An extract of brown algae, *Padina arborescens* (PAE; 0.5%, *w*/*w*), was proved to reduce the blood glucose, glycosylated hemoglobin, and plasma insulin levels, as well as plasma TC, LDL-C, TG, and FFA levels, and improve glucose tolerance in a 6-week mouse experiment. These may be modulated through the PAE-caused significantly lowered hepatic activities of glucose-6-phosphatase and phosphoenolpyruvate carboxykinase and increased glucokinase activity [57]. As an alginate modifier, 2% calcium alginate (Ca-Alg) was found can significantly reduce the plasma TC in rats fed a high-cholesterol diet for 2 weeks, and this was induced by increased fecal excretion of bile acid and reduced intestinal reabsorption, evidenced by a notably lowered portal concentration of bile acid, which in turn stimulates bile acid synthesis and leads to a decrease in plasma cholesterol [58]. Polysaccharide from the sporophyll of a brown alga *Undaria pinnatifida* (AP) could reach a yield of 38.7% from its dry matter, which was composed of about 80% alginate and 20% fucoidan. The 1.7% AP supplementation obviously reduced total weight gain and fat accumulation and improved the serum lipid profile in HFD-rats, including TG, TC, and VLDL-C, which were all closely linked with increased fecal weight and reduced gastrointestinal transit time. Moreover, the hepatic lipid peroxidation was reduced, suggesting a protective action of the liver against HFD [59]. Alginate at 20 g/kg notably reduced hepatic cholesterol to 13.1 *μ*mol/g but did not influence serum lipids. However, the amidated alginate at 20 g/kg significantly decreased serum TC from 2.93 to 2.00 mol/L, TG from 1.66 to 0.92 mol/L, hepatic cholesterol from 17.5 to 5.9 *μ*mol/g, and total hepatic lipids from 67.4 to 51.7 mg/g in female HFD rats through significantly increased fecal concentrations of neutral sterols from 98.7 to 122.4 *μ*mol/g dry matter [60]. Apolipoprotein E-deficient mice fed a HFD plus either 1% or 5% fucoidan for 12 weeks showed a significant reduction of liver and adipose tissue weight, blood lipid, TC, TG, non-HDL-C, and glucose levels but increased plasma lipoprotein lipase (LPL) activity and HDL-C levels. Fucoidan also improved hepatic steatosis and lipid profile [61]. Fucoidan treatment also significantly improved the serum lipid profile 2 h after administration of poloxamer-407, which induces acute hyperlipidemia in mice [62]. From above evidences, polysaccharide extracts from brown algae could exert obvious hypolipidemic action; however, the effective dosage may have not yet been systematically studied.

### 3.7. Some Types of Prebiotics

Prebiotics are resistant to be hydrolyzed in the small intestine but are fermentable by commensal intestinal microorganism and, therefore, gives growth-promoting effects on beneficial microbes such as *Bifidobacterium* sp. and *Lactobacillus sp*. The major type of prebiotics includes inulin, fructooligosaccharides (FOSs), galactooligosaccharides (GOSs), xylooligosaccharides (XOSs), maltooligosaccharides (MOs), lactulose, lactulosucrose, fructans, resistant starch, etc. [63]. Soluble prebiotics are able to increase the viscosity of the digestive tract and the thickness of the unstirred layer in the small intestine and thus inhibit the uptake of cholesterol [64]. The hypotriglyceridemic effect of prebiotics is also believed to be due to a reduction in hepatic reesterification of fatty acids in addition to modulation of the expression of liver lipogenesis-related genes, resulting in lower hepatic secretion rate of TG [65]. In addition, the beneficial modulation of microorganism-induced metabolite variation including SCFAs may also contribute to the hypolipidemic effect of prebiotics. Inulin, FOS, and GOS are the most popular prebiotics used in food industries, including infant food.

#### 3.7.1. Fructooligosaccharides (FOS)

FOS are naturally bioactive compounds, stored in many common foods, such as banana, garlic, asparagus, onion, wheat, and rye, and consisted of glucose and fructose residues joined by *β*-1,2-glycosidic linkages. An early study found that FOS prevents serum lipid disorders and lowered the activity of fatty acid synthase in the liver of rats [66]. A later study found that when rats received 2.5 g/kg lipid emulsion supplemented with FOS, their plasma TG was significantly suppressed compared with ones without FOS, and this may be caused by enhanced fecal excretion of lipids [67]. A reduced hepatic lipogenesis and steatosis caused by FOS is regulated by a reduction of the activity of lipogenic enzymes, leading to the reduction of VLDL-C and TG secretion [68]. Either 340 or 6800 mg FOS/kg body weight/day yacon root FOS supplementation for 90 days was observed with a significant decrease in fasting plasma TG and VLDL levels in a diabetic rat model [69]. The results of a human study indicate that the FOS supplementation enhanced obviously the reduction of LDL-C and steatosis of patients, who had more exercise and a balanced diet [70]. A systematic review also supported this idea, and the most obvious reduction is plasma TC levels [71]. Another study found that 2 g/d FOS plus probiotic increased significantly serum HDL-C levels, but no significant reduction of TC and TG in elderly people with type 2 diabetes mellitus was observed [72]. However, another team evaluated the supplementation with short-chain FOS 10.6 g/d in mild hypercholesterolemic patients and reported no significant reduction in plasma TC concentrations [73]. From the above evidences, most studies approve the beneficial effects of FOS on hyperlipidemia; however, the inconsistent results suggested more efforts are required.

#### 3.7.2. Inulin

When male hamsters were fed HFD plus 8, 12, or 16% inulin for 5 wks, their serum TC concentrations were significantly reduced by 15%-29%, TG were significantly lowered by 40%-63%, and only 16% inulin specifically decreased VLDL-C, while LDL-C and HDL-C were not significantly altered. Further notable changes in the bile acid and hepatic lipid profile demonstrate that the lipid-lowering action of inulin is possibly due to an altered hepatic triacylglycerol synthesis and VLDL secretion and reduced reabsorption of bile acids [74]. Hypercholesterolemic rats had a significant decline in plasma LDL-C and a significant rise of HDL-C levels compared to the control after 4 weeks of inulin intake, and this is associated with more excretions of fecal lipid and cholesterol [75]. Although enough positive data of lipid-lowering effects have been observed in animals, a relatively high dose of inulin had to be applied. There are also some human studies evolving. Unbalanced serum lipid levels accompanied by diabetes mellitus were found to be alleviated by inulin supplementation, including a significant reduction in TC by 12.90%, TG by 23.60%, and LDL-C by 35.30% and a rise of HDL-C by 19.90% [76]. In another 4-week trial, subjects consumed 50 g cereal,which includes 18% of inulin, showed significantly decreased plasma TC, TG, and total facultative anaerobes and increased bifidobacteria, while the weight, fecal bile acid excretion, SCFAs, and fecal pH were not notably altered. Besides, there is a remarkable finding that the modulation of serum lipids was negatively correlated with bifidobacteria number and positively related to the output of secondary bile acid [77]. A review that included 9 inulin or oligofructose supplementation in human volunteer studies found 3 of them exhibited significant reductions in TG, 4 of them showed modest reductions in TC and LDL-C, while only 2 of them reported with no effects [78]. Considering these studies have been conducted in both normal and moderately hyperlipidemic subjects and the dose and experimental duration were all varied, it is thus reasonable that their results are inconsistent. Besides several important animal studies that have supported the idea that the lipid-lowering effects of inulin and oligofructose originate mainly from the inhibition of fatty acid synthesis in the liver, however, this pathway is relatively inactive in man for human seldom consumes a high-carbohydrate diet. There are also other controversial results. Pedersen et al. [79] investigated the effect of 14 g/d of inulin consumption on blood lipids in young healthy women having a limited fat intake for 2 months and did not find any significant differences between the two groups. Similarly, 17 healthy subjects after 6 months of daily administration of inulin and oligofructose without modifying their way of life exhibited no effect on serum TG levels and hepatic lipogenesis and only a slight decrease in TC and LDL-C levels [80]. These two results may be explained by a recent study, in which the hypolipidemic effect of inulin differed depending on dietary fat content (5% versus 20%). This study also suggested that lipid-lowering action of inulin mainly comes from the increased excretion of total lipid and neutral sterol [81].

#### 3.7.3. Resistant Starch (RS)

Resistance for digestion of RS originates from its compact structure and partial crystalline structure, which has been regarded as a kind of prebiotics to be beneficial for health, including lose weight, reduce lipids, and prevent intestine diseases. Compared with the wheat starch group, 20% RS significantly induced the cecal hypertrophy by 2.4 times and accumulation of SCFAs, while the cholesterol absorption was reduced from 47% to 14%. RS also effectively reduced the plasma TC by about 30% and TG by about 25%. Moreover, there were apparent lower concentrations of TC and TG (-50%) in the livers of RS-fed rats, too [82]. Similarly, the plasma TC, VLDL-C, and LDL-C concentrations were all significantly reduced and fecal total bile acid concentration, total SCFAs, and acetic acid were all significantly higher when rats received 0.5% cholesterol plus 15% bean RS. This also suggested that the hypolipidemic effect of RS may be attributed by its action against absorption and fermentation effect in the intestine [83]. RS has been well characterized for its glycemic control properties, which may also have impact on lipid metabolism. Besides the doubled HDL-C concentration, the results of 2 g/d RS administration in type 2 diabetic rats showed that blood glucose level and TC and TG concentrations were all significantly reduced (*p* < 0.01) [84]. On the contrary, another experiment using 12 8-week-old male pigs consumed a synthetic western diet with (10 g/RS/day) or without potato starch reached different lipid profiles. Although the serum lipids including TC, LDL-C, VLDL-C, and TG were similar, HDL-C particles were obviously higher by 28% and fasting serum glucose was lowered by 20% in the RS group [85]. Healthy overweight patients were given either 24 g/d of RS or regular corn starch for 21 d in addition to their regular meals. Although RS resulted in no significant changes in their weight or other physical parameters, there were significant lowering effects of plasma TC, LDL-C, and the mean fasting serum glucose levels in subjects supplemented RS [86]. These studies all suggested that the consumption of RS may be beneficial in lipid management strategies in addition to lowering blood glucose, but the connection with SCFA production and glycemic effect still needs further study. In order to prove RS as a novel therapeutic agent of hyperlipidemia especially in diabetic patients, controlled trials with larger sample sizes and longer duration both in animal and human are required.

### 3.8. Gum

A study found that guar gum significantly lowered the fecal lipid digestibility and the intestinal conjugated bile salts (*p* = 0.0001) for both control chicken and sterilized one [87]. One milligram partially hydrolyzed guar gum (PHGG) significantly decreased the TC, LDL-C, TG, and VLDL-C and delayed the formation of arterial thrombus in rat fed HFD. In addition, the increased Bax and decreased Bcl-2 and HSP-70 protein expression were found to be balanced by PHGG in the arteries of HFD hamsters [88]. Diet containing either 5%, 10%, or 20% guar gum was fed to diabetic rats for a month. Although diabetes elevated serum lipids in all rats within 2 weeks, the guar gum diet significantly reduced the plasma TC, TG, and LDL-C levels as well as the atherogenic index, suggesting that guar gum was effective in the treatment of hyperlipidemia in diabetes rats [89]. Guar gum treatment also decreased markers of the metabolic syndrome, including body weight, adipose weight, TG, glucose, and insulin levels in a dose-dependent manner in HFD mice [90]. Guar gum of 3 different viscosities was assessed in male rats fed HFD for 3 weeks indicating while all guar gum can reduce TC, liver steatosis, and blood glucose levels, only the medium one was most effective in preventing the diet-induced hyperlipidemia and liver steatosis [91]. The effect of 6 g PHGG in yogurt on postprandial plasma lipid concentrations was tested in 11 healthy male adults. Results indicated that the supplementation significantly suppressed the incremental peaks and areas of postprandial plasma TC and TG [92]. After 10 wks of HFD plus 5% guar gum in rats, the fat mass percentage, epididymal fat pad weight, and the liver lipid concentrations were all significantly lower than the controls [93]. However, there is also controversial result. A study reported that supplementation of dietary fiber at the 5% level for 3 weeks, including cellulose, guar gum, PHGG, glucomannan, highly methoxylated pectin, and guar gum, on normal rats induced no significant effect on the serum lipid levels [94]. Considering this study is referred to only normal subjects, most other animal study showed that guar gum and PHGG are effective lipid-lowering agents; however, the effect on human still needs more experimental data.

### 3.9. Hydroxypropylmethylcellulose (HPMC)

HPMC is also a food gum, which shares many common characteristics with soluble fibers, such as high viscosity, and has been widely adopted in the food and medicine industries as emulsifier, diaphragm, suspender, thickener, dispersant, and stabilizer. It is a nonfermentable dietary fiber and has also been also demonstrated with modulation effect of lipid metabolism. In a trial, after a baseline period, 51 mild-to-moderate hypercholesterolemic men were randomly divided into two groups to consume 5.0 g/d HPMC with or between meals for 2 weeks. In the between-meal group, TC was reduced by 5.1%, LDL-C by 7.7% (both *p* < 0.01), while in the with-meal group, reductions were 8.3% for TC and 12.8% for LDL-C (both *p* < 0.01), which suggested that HPMC has a better lipid-lowering effect when taken with meals [95]. In another trial, HPMC were given differently in dose 3, 5, or 10 g/d of low, moderate, moderately high, or high viscosity to hypercholesterolemic patients. Results showed that all HPMC could reduce LDL-C ranging from 6.1% to 13.3%, and the reduction of TC and non-HDL-C was associated with that for LDL-C, but HDL-C, TG, and apolipoprotein B were not significantly altered [96]. This team also conducted another experiment to examine the lipid profile of 2.5 g × 2/d HPMC consumed subjects with hypercholesterolemia after at least 4 weeks of statin therapy. Results showed that HPMC consumption resulted in significantly larger reductions in TC (10.9 vs. 3.5%), non-HDL-C (12.8 vs. 2.9%), LDL-C (15.7 vs. 5.1%), and Apo B (8.7 vs. 3.9%), which support the view that HPMC is an effective adjunct to statin therapy in patients with primary hypercholesterolemia [97]. Another study found that rats with a HFD plus 5% HPMC had obviously reduced epididymal fat pad weight and liver lipid concentrations. The HPMC group also had an obvious higher *ex vivo* palmitate oxidation in muscle compared with the same dose of a fermentable fiber, guar gum, implying a higher oxidation capacity to FAs, which demonstrate that HPMC can reduce the adiposity and hepatic steatosis induced by HFD, and this ability does not correlate with fermentability [93]. Another study found that 6% HPMC could significantly reduce 55% of body weight gain, 13% of liver weight, and 45% of plasma LDL-C concentration of mice compared to the same dosage of microcrystalline cellulose- (MCC-) fed mice through upregulating genes related to fatty acid oxidation and synthesis of cholesterol and bile acids and downregulating genes related to oxidative stress, triglyceride synthesis, and polyunsaturated fatty acid elongation in the liver [98]. The above evidences suggest that 5 g/d in human diet or 5-6% of HPMC in animal feeding could both exert relative strong lipid-lowering effect; more research will be required to define the roles of viscosity on the lipid-modulating effects of HPMC and the dosage relationship between human and animals.

### 3.10. Whole Grain or Arabinoxylan

Whole grain, referring to grain with unpeeled bran, which includes wheat, rye, and oat, owns high content of both soluble and insoluble DFs, which comprises mainly of arabinoxylans and glucans. A multicompartmental metabolomics study comparing whole grain rye with added rye bran with refined wheat in pig found that rye bran induces lower levels of linoleic acid-derived oxylipins and TC in the plasma [99]. Compared to 0% wheat bran (WB), 10 or 20% of WB induced obvious decrease in TC and HDL-C, while 5, 10, or 20% WB induced similar reduction in PL and TL in a dose-dependent manner [100]. In another study, hamster feeding experiment found that 5 g/kg of wheat bran arabinoxylans (Axs) lowered plasma TC and LDL-C concentrations and increased the output of TL, TC, and bile acids through reducing the activity of HMG-CoA reductase and increasing the activity of CYP7A1 in the liver as well as concentrations of SCFAs in the gut [101]. These results indicated the AXs lower the plasma lipids through promoting the excretion of fecal lipids, regulating the lipid metabolism related genes, and producing more colonic SCFAs. Besides a significant reduction in TG, LDL-C, and an increase in HDL-C, a wheat fermented powder also caused a significant variation of important antioxidant biomarkers in a rabbit feeding experiment [102].

### 3.11. Chitosans

There are multiple studies reporting the hypolipidemic effect of chitosan in animal models. It was suggested that subacute toxicity of chitosan was small and the observed adverse effect level was considered to be over 2,000 mg/kg in rats [103]. Supplementation of 5% chitosan for 12 wks in rats could significantly decrease serum concentrations of TC, LDL-C, and hepatic levels TC, TG and increase the output of fecal bile acids, but the plasma levels of TG and HDL-C were considered unaltered. In addition, the result of RT-PCR showed that chitosan could reverse the reduction of LDL receptor mRNA levels, which led by the intake of saturated fat and cholesterol [104]. 250-1000 mg/kg chitosan oligosaccharide (COS) administration of mice caused a significant reduction of serum TC and LDL-C and a significant increase in peritoneal macrophage-derived ^3^H-cholesterol in liver and bile as well as in feces, which suggested a positive role for COS in reversion of the cholesterol transport. In addition, the observed lipid-lowering action was in a dose-related relationship of the modulation of hepatic protein expressions of CYP7A1, SR-BI, and LDL receptor (LDL-R) by COS [105]. Six weeks of 5% chitosan supplement was found to significantly decrease the body weight gain and the lipids level both in the plasma and liver, while it was found to increase the output of fecal fat and cholesterol and hepatic lipoprotein lipase activities of HFD rats compared with rats only fed a HFD, which suggested that chitosan improves obviously the hypercholesterolemia in rats through reducing the absorption of fat and cholesterol [106]. Lower plasma TC, LDL-C, VLDL-C, and apolipoprotein B (Apo B) concentrations as well as higher HDL-C and no significant difference in TG or glucose levels were observed in rats fed a diet containing chitosan for 2 wks. In addition, rats fed the chitosan diet had a changed composition in VLDL particles as evidenced by increased TG percentages and core lipid proportions and decreased free cholesterol, cholesteryl ester, phospholipid, and the surface lipid proportions, suggesting that chitosan has a great influence on the VLDL particle formation and regulation of lipoprotein metabolism in rats [107]. Three studies are involved in the evaluation of different MW of chitosan. Among 21, 46, and 130 kDa, the medium one was the most effective one to inhibit pancreatic lipase activity *in vitro* and to reduce the serum TG and thus, it was fed to mice together with a HFD for 20 weeks. It prevented the increase of body weight, mainly the accumulation of white adipose tissue and liver lipids including TC and TG, and further increased the fecal bile acid and fat. The results suggested that the hypolipidemic action of this chitosan may be through increasing the excretion of fecal fat and bile acid caused by its binding activity and through inhibition of pancreatic lipase activity and subsequently decrease the absorption of dietary lipids from the small intestine [108]. Similarly, another study compared the lipid-lowering activities of high (712.6 kDa) and low (39.8 kDa) MW chitosan in rats fed HFD for 8 weeks. The low one was more effective in decreasing the body weight gain, serum TC, and LDL-C, as well as decreased liver TG. The activities of liver and serum lipoprotein lipase and fecal fat level were also higher than the high MW group [109]. However, the result is quite opposite in streptozotocin- (STZ-) induced diabetic rats, which reported that rats fed with both high MW (100 kDa) and low MW (14 kDa) chitosan had increased HDL-C, whereas significantly decreased plasma glucose and TC and increased fecal cholesterol excretion were observed only in diabetic rats fed with high MW chitosan [110]. From these results, we could draw a primary conclusion that the MW of chitosan strongly affect its hypolipidemic effect and the ideal MW is between 21 and 100 kDa. Moreover, compared with untreated chitosan, the reducing effects of medium-milled chitosan on serum TG, TC, and LDL-C and liver TG and TC were all increased by about 10% [111].

There are just a few reports about chitosan's lipid-lowering effect in humans. A meta-analysis of 6 RCTs with 416 hypercholesterolemia patients concluded that it has a significant effect on TC (-0.3 mmol/L (11.6 mg/dL); *p* = 0.002) but not on LDL-C, HDL-C, or TGs [112]. However, several other studies showed that it could exert an effect on LDL-C. One of them declared that the dietary chitosan could reduce serum TC levels by 5.8–42.6% and LDL-C levels by 15.1–35.1% [113]. A 12-week trial found an overall treatment effect of a 40 kDa chitosan from the placebo group. The 2.4 g once-daily group reducing LDL-C by 16.9% showed the best, even better than the same dosage but separately administrated group, which reduce the LDL-C only by 9.7%. But there were 29 mild adverse events reported by 23% patients related to the chitosan treatment, including constipation and diarrhea [114]. The EFSA suggested that evidences from chitosan indicated a small, but statistically significant effect on the reduction of both TC and LDL-C levels, with no effect observed on HDL-C. The panel suggested further that in order to achieve this effect on blood lipids, 3 g/d chitosan should be applied [115]. Taken together, chitosan possesses the ability to lower lipids but may cause some side effects; therefore, more controlled clinical trials of a longer duration are essential to assess the dose-hypolipidemic effects besides the evaluation of its adverse effects.

### 3.12. Other Insoluble DFs

Different insoluble dietary fibers cholestyramine, chitosan, and cellulose have been assumed with high, intermediate, and low ability to bind with bile acids, respectively. Especially, the cholestyramine has long been clinically applied as a cholesterol-lowering, bile acid-binding drug. A study reported that the consumption of 7.5% of either one of the three dietary fibers showed similar hypolipidemic activity in mice fed a diet containing high levels of fat and cholesterol; however, cholestyramine showed the best ability to deplete the hepatic cholesterol and this may be induced by a decrease of cholesterol absorption efficiency and an increase of fecal bile acid and cholesterol excretion, which is caused by its high capacity to bind with bile acids. However, chitosan or cellulose reduced only the food intake including cholesterol, but the impact is neither on intestinal cholesterol absorption nor on the output of fecal bile [116]. Lignin extracted from olive stones was found to be able to bind significantly more bile acids than any other fraction, and the capacity is similar to that of cholestyramine, especially when cholic acid was applied *ex vivo* [117]. From these evidences, the hydroinsoluble DF may contribute more to affect the absorption of cholesterol.

### 3.13. Combination of Soluble and Insoluble DFs

Okara owns a high dietary fiber content of 54–55%, mainly IDF but also SDF content. High-cholesterol-fed rats supplemented with enzymatically treated okara showed a significant reduced TG levels both in serum and in the liver but a higher TL, TG, and bile acid (*p* < 0.001) in the feces. The improved intestinal transit by increasing fecal bulk, the decreased pH, and increased SCFA production indicated that this okara exerts a potential prebiotic effect [118]. Another study compared the effects of cellulose and pectin on the metabolism of lipid and carbohydrate in rats for 6 wks. The TC level in plasma was significantly lower while plasma HDL-C was significantly higher in 5% of the pectin group while rats fed the same dosage of cellulose had lower contents of TC and TG in the liver. However, a significantly lower plasma glucose was observed in 2.5% of cellulose plus 2.5% of the pectin group [119]. These results suggested that a diet containing fiber may be a possible adjuvant treatment for correcting some disturbances of metabolism, but IDF and SDF should be considered separately for their different mechanism to exert the balancing effect. However, in some occasions, they need to be applied in a combination to maximize this effect.

### 3.14. Modifiers of DFs

It has long been predicted that chemical modifications may increase the cholesterol-lowering effect of polysaccharides. Hydrophobic derivatives of highly alkyl or acyl group-substituted pectin, chitosan, and cellulose, all confirmed this prediction, which have high binding capacity to cholesterol and thus left fewer cholesterol for enterohepatic circulation [120]. A study also found that hydrophobic amidated pectins significantly altered cholesterol homeostasis in rats and amidation of pectin decreased its fermentability to produce lower cecal SCFAs in rats [121]. The administration of Cymodocea nodosa sulphated polysaccharide (CNSP) exhibited a better serum lipid level by decreasing lipase activity of obese rats compared with untreated polysaccharides. Additionally, CNSP administration to HFD rats induces further antioxidant activity [122]. A modifier was further prepared by this team using highly methoxylated citrus pectin with a degree of 60% substitution of N-octadecylamine, which significantly decreased the concentrations of cholesterol in hepatic tissue and triacylglycerols in serum. The feeding experiment showed its potential to substitute typical antilipidemic drugs at a low dose and used for a period shorter than 3 months [123]. In another study, the interaction of chitosan and its two derivatives with plasma leptin, glucose, insulin, and total cholesterol and further interaction with mRNA expression of adipocytokines were investigated in a diet-induced insulin-resistant rat model. The results proved that all the three substances not only lowered the level of plasma leptin, glucose, insulin, and TC *in vivo* but also downregulated the mRNA expression of leptin and resistin and up-regulated the mRNA expression of adiponectin and PPAR-*γ in vitro*. In addition, the two chitosan derivatives exhibited better regulating effect [124].

## 4. Mechanisms of DFs to Affect Lipid Metabolism

**Figure 2:**
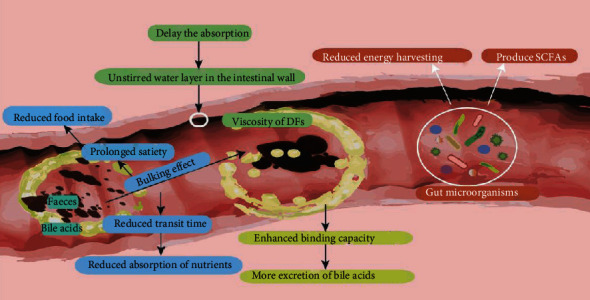
Possible positive effects of typical dietary fibers on hypolipidemic process.

Five major mechanisms are believed to be responsible for the antihyperlipidemic benefits of DFs, including low levels of energy, bulking effect, viscosity, binding capacity, and fermentation, which are summarized in Figure 2. Foods rich in DFs include whole grain products, legumes, and fruits classified as low GI foods, which refer to a relatively low glycemic effect compared to an equal amount of available carbohydrate (usually from white bread or glucose). Fully fermentable RS, for instance, has been estimated to contribute about 8.8 kJ/g, whereas glucose contributes 17 kJ/g [125]. Some fibers, generally referred to as IDF, provide bulking effect, hence increasing stool mass, alleviating constipation, and improving regularity. Meanwhile, most of the SDF is associated with a great water-holding capacity. Lactulose, for example, has long been considered a laxative and proved to be effective in multiple constipation intervention studies. The increased stool weight is due to the physical presence of DF as well as the water held inside the fiber matrix, such as cellulose and lignin; although are mostly not fermentable in the colon, they can effectively increase fecal bulk by their particle formation and water-holding capacity. Due to the increased bulk and water content, the nutrients in the intestine are diluted, including sugar and lipids, and their migration to the intestinal walls also slows down. The IDFs are also associated with decrease in intestinal transit time that helps reduce the absorption time of these sugars and lipids. As a result, they can reduce the absorption of macronutrients, especially carbohydrates and cholesterol either by delaying gastric emptying or by shortening small intestinal transit time, in addition to a reduced glycemic response, which could further assist the reduction in insulin stimulation of hepatic cholesterol synthesis [126]. These intrinsic properties of DFs that relate to bulking and viscosity effects also promote prolonged satiety and reductions in food intake, which accounts for another important mechanism of lipid lowering [20]. In addition to the fiber's viscosity, the ability of DFs to bind to bile to disturb the reabsorption of bile salt from the small intestine is another factor that leads to synthesis of new bile acids from cholesterol and hence reducing blood cholesterol levels and lipid-lowering effect [127]. Since the production and excretion of bile acids represent the major pathway of cholesterol removal from the body and the biosynthesis of bile acids in the liver is modulated through a series of positive and negative feedback mechanisms, the increased fecal removal of bile acids reduces the amount of bile acids present in the plasma. 5% guar gum, for an example, significantly lower lymph flow and lymphatic lipid transport and thereby diminishes lipid transport by means of its physicochemical properties related to water behavior in the intestine [128]. DFs undergo a fermentation process to produce short-chain fatty acids (SCFAs), including propionate, acetate, and butyrate. The concentration of both acetate and propionate is reported to be increased more than 2-fold in the rats' portal plasma after the oligofructose intake. However, the involvement of SCFAs is, as for lipid-lowering effect, makes it difficult to draw a conclusion with complete identity, as they have an antagonistic effect: propionate has been reported to inhibit fatty acid synthesis [129], whereas acetate is a lipogenic substrate. Despite a large part of SCFAs absorbed by the host, which accounts for about 10% of daily energy supply [130], gut microorganisms rely fast entirely on the energy from fermentation process. This fermentation process reduces the total energy supply of the body, thus balancing the lipid metabolism in hyperlipidemic patients. From this perspective, this fermentable property of DFs exerts beneficial effect on balancing lipid metabolism compared to other nutrients being directly digested in the small intestine. Studies also revealed that SCFAs could cause satiety and inhibit cholesterol synthesis [131, 132]. We will discuss the relationship between lipid metabolism and SCFAs more specifically below for its complexity, focusing on the molecular mechanism. Each kind of DF distinguishes from one another in these five properties for sure. For instance, the different molecular weights of *β*-glucan were found to have no impact on its total fermentation products but affect its viscosity, which has further impact on its binding capacity to bile acid, absorptive layer in the small intestine, and transit time [133]. However, more or less of these five major properties of DFs indicates that DFs play a positive role in the hypolipidemic process.

## 5. Molecular Mechanism of the Hypolipidemic Effect of DFs

### 5.1. 3-Hydroxy-3-methylglutaryl Coenzyme A (HMG-CoA) Reductase

HMG-CoA reductase, officially abbreviated as HMGCR, is the rate-controlling enzyme of the mevalonate pathway, which is the metabolic pathway producing cholesterol and other isoprenoids. In mammalian cells, this enzyme is suppressed by cholesterol and degradation of LDL via the LDL receptor as well as oxidized species of cholesterol. Meanwhile, its competitive inhibitors will induce the hepatic expression of LDL receptors, which in turn increases the catabolism of plasma LDL-C and lowers the plasma concentration of lipids. This enzyme is thus the target of the widely used cholesterol-lowering drugs, such as the statins. An early study found that the addition of 5% chitosan to sterol diet suppressed the increase of plasma and liver cholesterol by 54% and 64%, respectively, and this is correlated with the chitosan-modulated reduction of HMG-CoA reductase activity by four times, compared to high-sterol diet-alone rats [134]. Most of the later studies referring to the hypolipidemic mechanism of dietary fiber come to a similar finding that HMG-CoA reductase activity was downregulated [38, 53, 62, 83, 101, 135], which indicates the possibility of DFs as adjunct to traditional lipid-lowering drug or adjuvant therapy for hyperlipidemic patients.

### 5.2. LDL Receptors

As discussed above, the upregulated expression of LDL receptors will increase the decomposition of LDL-C, which means a balance of lipid metabolism in hyperlipidemic patients. A study found that hepatic protein expressions of LDL receptor were improved in a dosage-dependent manner in chitosan oligosaccharide- (COS-) administered mice. In addition, the expression of scavenger receptor BI (SR-BI), which plays a critical role in cholesterol uptake from plasma to the liver, was also upregulated in a dose-dependent manner of COS supplementation mice, while the main transporters for transferring cholesterol to plasma HDL, the level of ABCA1 and ABCG1 remains unchanged, which also correlates with the unchanged HDL-C level [105]. However, barley bread enriched with HPMC was found to downregulate the expression of the ABCG5 gene [38]. Fucoidan was found to attenuate the hepatic expression of mature SREBP-2 protein with a subsequent decrease in hepatic HMG-CoA reductase mRNA expression and an increase in hepatic LDL receptor mRNA expression, indicating that fucoidan improves serum lipid levels by regulating the expression of key enzymes of TC and LDL-C metabolism in the liver through modulation of SREBP-2 [62].

### 5.3. Cytochrome P450 7A1 (CYP7A1)

Cytochrome P4507A1 (CYP7A1) also known as cholesterol 7-alpha-monooxygenase or cholesterol 7 alpha-hydroxylase, an important member belonging to the cytochrome family, has an important role in cholesterol metabolism because it catalyzes the conversion process from cholesterol to 7-alpha-hydroxycholesterol, the first and rate-limiting step in bile acid synthesis. The activation of CYP7A1 leads to an increase of bile acid biosynthesis thus decreasing the concentration of cholesterol. Bile acids provide feedback inhibition of CYP7A1 by at least two different pathways, one involving the farnesoid X receptor (FXR) and small heterodimer (SHP) as well as liver receptor homolog (LRH-1) and another involving inflammatory cytokines, including TNF-*α* and IL-1*β* [136]. CYP7A1 is upregulated by the nuclear receptor liver X receptor (LXR) when cholesterol levels are high and downregulated by sterol regulatory element-binding proteins (SREBP) when plasma cholesterol levels are low [137]. The activity of CYP7A1 in the liver was significantly increased by *β*-glucan from both barley and oats compared with the control [135]. The extreme lipid-lowering action of pea proteins plus apple pectin was also found to be regulated by CYP7A1 and sodium/bile acid cotransporter (also known as the Na^+^-taurocholate cotransporting polypeptide (NTCP) or liver bile acid transporter (LBAT)) [53]. The lowered TC and LDL-c concentrations and increased excretions of TL, TC, and bile acids caused by wheat bran arabinoxylans were also found to be modulated by increased activity of CYP7A1 in the liver [101]. Similarly, the mRNA level of CYP7A1 of the HMPC group was found upregulated by 1.9-fold and the expression levels of SREBP-1c and stearoyl-CoA desaturase (SCD-1) were downregulated by 2- and 5-fold, respectively, relative to the control group [98]. Hepatic gene expression profiles demonstrated that polysaccharide from *Lycium barbarum* (LBP), a well-known Chinese traditional herbal medicine, can activate the phosphorylation of AMPK, suppress nuclear expression of SREBP-1c, and decrease protein and mRNA expression of lipogenic genes *in vivo* and *in vitro*. Moreover, LBP significantly elevated uncoupling protein-1 (UCP1) and peroxisome proliferator-activated receptor coactivator-1 (PGC-1) expression of brown adipose tissue [138]. The fecal effect of glucan also could activate CYP7A1, which catalyzes the rate-limiting step in the biosynthesis of bile acids from cholesterol, leading to an upregulation of bile acid synthesis from plasma cholesterol and thus lowering the circulating LDL-c levels [139]. Most of the other studies also found that CYP7A1 is an important target of DF's lipid-lowering action [38, 105].

### 5.4. MAPK Signaling Pathway

The MAPK signaling pathway was found to regulate the expression of CCAAT-enhancer-binding proteins *α* (C/EBP*α*) and peroxisome proliferator-activated receptors *γ* (PPAR*γ*) mRNA during adipogenesis process in 3T3-L1 cells and thus play an important role in the process of lipid metabolism. A water-soluble extract of *P. binghamiae thalli* (PBEE) including water-soluble polysaccharides is able to inhibit preadipocyte differentiation and adipogenesis in a dose-dependent manner, which is caused by the decreased the expression of PPAR*γ* and fatty acid-binding protein aP2 [140]. Similarly, mice fed a high-fat diet supplemented with 10% guar gum for 12 weeks also induced correction of metabolic abnormalities caused by PPAR*γ* repression, subsequently increasing mitochondrial uncoupling protein 2 (UCP2) expression and AMP/ATP ratio, leading to the activation of AMPK [141]. Fucoidan could decrease the lipid accumulation through inhibiting the expression of both early C/EBP*α* and PPAR*γ* and late aP2 adipogenic transcription factors, which play a crucial role for adipocyte development. Moreover, fucoidan also inhibited the early activation of p38 MAPKs, extracellular signal-regulated kinases p-ERK1/2, and Jun N-terminal kinase p-JNK activity in a dose-dependent manner [142].

### 5.5. Other Lipid Metabolism-Related Genes

A study found that fucoidan decreases the expression of FAS and ACC mRNA with only moderate inhibitory effect on SREBP-1c mRNA expression in both HepG2 hepatocytes and the mouse liver [62]. Our latest work also focused on the molecular mechanism of the antihyperlipidemic effect of rice bran polysaccharides (RBP) in high-fat diet mice. Besides significantly reduced weight and liver and fat pad weight, improved lipid profile in the plasma and recovered fat liver lesion were observed under the protection of RBP. Microarray analysis revealed that RBP could result in a regulation of over 150 genes, including multiple genes involved in the hepatic lipid metabolism like Sult3a1, Sult3a2, several CYPs, Acnat2, Acot6, SERPINA3, SERPINA6, RORA, and several APOs. IPA database suggested further that NF-*κ*B may play a vital role in the lipid-lowering effect of RBP. Real-time quantitative PCR and western blot confirmed that RBP affect several lipid metabolism target genes including PPAR-*α*, PPAR-*γ*, PPAR-*δ*, SREBP-1C, FASN, ACC, SIRT, and CD36 [143]. Another microarray test compared the hepatic expression level of gene between HPMC supplementation and only HFD-fed rats, and the results overlapped with our results to a large extent: Serpina6, Aqp8, Hsd17b7, Nsdhl, Tm7sf2, and Cyp51. There are also some genes involved in fatty acid *β*-oxidation, such as Ehhadh and Acacb, and the elongation of very long-chain fatty acid-like 2 (Elovl2), sterol-C4-methyl oxidase-like (Sc4 mol), and patatin-like phospholipase domain-containing 2 (Pnpla2), which is involved in triglyceride breakdown by regulating adipose triglyceride lipase, was all upregulated [98]. DNA microarray analysis and q-PCR also demonstrated that fucoidan induces differential expression of genes encoding proteins involved in lipid metabolism, energy homeostasis, and insulin sensitivity, by activating PPAR*α*, inactivating Srebf1, and affecting LPL activity in HFD-fed ApoEshl mice [61]. Another study evaluated gene expression profiles in the small intestinal mucosa of db/db mice fed with PHGG. DNA microarray and real-time PCR analyses reported that PHGG upregulated the expression of 9 genes, including Oas3, Oas1g, Duox2, and Nlrc5, potentially related to host defense functions, and downregulated the expression of 8 genes, including sterol O-acyltransferase (Soat1), which is involved in cholesterol esterification and absorption, in the small intestine [144]. The expression levels of lipid oxidation gene Acox1, glycogen synthesis genes, GS2 and GYG1, and insulin-induced genes, Insig-1 and Insig-2, were significantly upregulated while fatty acids and triglyceride synthesis and metabolism-related gene SREBP-1, fatty acid synthesis gene (Fads1), and gluconeogenesis gene G6PC1 were greatly downregulated in RS-administrated diabetic rats [84].

### 5.6. SCFAs

**Figure 3:**
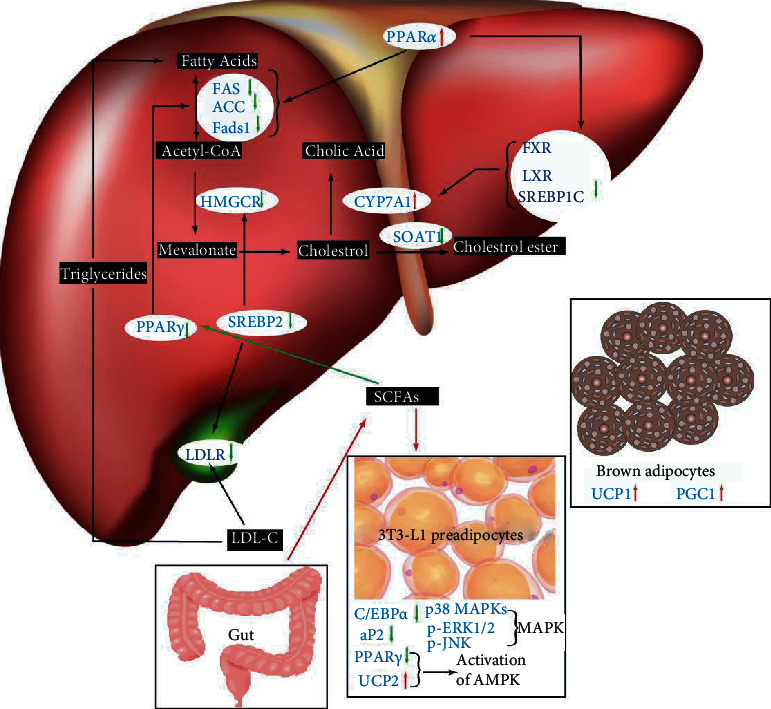
Possible molecular mechanism of dietary fibers on lipid lowering.

Given that SCFAs also count for a part of lipids and energy, food rich in DFs seemed to stimulate hyperlipidemia through harvesting the metabolites. But epidemiological study results suggest that they prevent it rather than promote it. Propionate, for instance, at the concentration of 0.6 mmol/L, could decrease the expression level of fatty acid synthase mRNA in cultured hepatocytes and thus considered a mediator having an antilipogenic property [68]. In addition, a 2-fold concentration of propionate at the portal vein of rats supplemented with fructan compared to controls selectively decreased the transition of acetate into total lipids [145]. A study found that the fluxes of SCFAs rather than concentrations reversely correlate with biomarkers of the metabolic syndrome in an animal experiment, including body weight, adipose weight, and TG [90]. The same team suggest further that SCFAs induce a PPAR*γ*-mediated switch from lipid synthesis to consumption. Oral sodium acetate, sodium propionate, and sodium butyrate supplementation prevented and reversed HFD-induced metabolic abnormalities in mice by decreasing PPAR*γ* expression and activity. This increased the expression of mitochondrial uncoupling protein 2 and increased the ratio of AMP to ATP, leading to an acceleration of the hepatic and adipose oxidative metabolism via AMPK. The mediator function of PPAR*γ* could also be proved by PPAR*γ*-absent mice, who exhibited no protective effect of the same supplementation. These results demonstrated that adipose and hepatic PPAR*γ* are critical mediators of the beneficial effects of SCFAs on the metabolic syndrome, indicating that SCFAs may be used therapeutically as cheap and selective PPAR*γ* modulators [146]. Studies also revealed that SCFAs could cause satiety via increased production of GLP-1 and PYY by stimulating FFARs and inhibit the rate-limiting enzyme of cholesterol HMGCoA reductase to inhibit cholesterol synthesis [131, 132]. Guar gum-induced obvious increase of peripheral glucose clearance also may be mediated by the SCFAs, for they are responsible for the change of colonic hormone glucagon-like peptide-1(GLP-1), which also has impaction on the lipid metabolism [141]. We summarized these possible molecular mechanisms of typical dietary fibers affecting the lipid metabolism in Figure 3.

## 6. Future Perspectives

Other than the direct effect of DFs on hyperlipidemia, the site of action-targeted drug delivery system using polysaccharides as package, such as pectin, dextran, gum, alginate, inulin, and konjac glucomannan, has attracted increased attention because this method could increase the bioavailability of the drug at the target site and meanwhile reduce the side effects. A study suggested that the employment of ultrafine redispersible spray dried emulsion with pectin as a carrier to form a delivery method for atorvastatin calcium holds a promising approach for an enhanced antihyperlipidemic effect for the widely used drug [147]. Another test that found an optimized formulation using glycyrrhetinic acid-modified chitosan as a liver-targeted carrier of atorvastatin showed increased plasma concentration, and the accumulation in the liver was nearly 2.59 times more than the plain drug nanoparticles [148]. Pharmaceutical and pharmacological indicators suggested that the proposed strategy can be successfully utilized for liver targeting of therapeutics.

There has been increasing interest in the effect of dietary fiber, on lowering the blood lipid concentration. There are various mechanisms by which serum and hepatic lipids are reduced by dietary fiber: binding to bile, viscosity, and bucking in the small intestine caused the suppression of glucose and lipid absorption, increased production of SCFAs, and modulation of lipid metabolism-related genes. In addition, dietary fibers, classified as the seventh nutrients, are generally considered safe, but overconsumption could cause intestinal discomfort. From the above evidences, dietary fibers could be used as alternative supplements to exert health benefits, including lipid-lowering effects on humans. However, more clinical evidence is needed to strengthen this proposal and its fully underlying mechanism still requires more investigation. Only if we fully understand the mechanism and dose relationship of each kind of DFs we are able to apply them in the intervention of hyperlipidemic patients.

## Abbreviations

SCFA: Short-chain fatty acids
CVD: Cardiovascular diseases
PL: Phospholipid
TL: Total lipid
TC: Total cholesterol
TG: Triacylglycerols
LDL-C: Low-density lipoprotein cholesterol
HDL-C: High-density lipoprotein cholesterol
VLDL: Very low-density lipoprotein
FOS: Fructooligosaccharides
PAI-1: Plasminogen activator inhibitor-1
fVII: Factor VII
HPMC: Hydroxypropyl methyl cellulose
EFSA: European Food Safety Authority
KGM: Konjac glucomannan
FFAs: Free fatty acids
GOS: Galactooligosaccharides
XOS: Xylooligosaccharides
MO: Maltooligosaccharides
SDF: Soluble dietary fiber
IDF: Insoluble dietary fiber
RS: Resistant starch
GG: Guar gum
PHGG: Partially hydrolyzed guar gum
AX: Arabia xylan
APO: Apolipoprotein
ACC: Acetyl-CoA carboxylase
LDLR: Low-density lipoprotein receptor
Dgat1: Diacylglycerol acyltransferase1
SR-BI: Scavenger receptor-BI
ABCA: ATP binding cassette subfamily A
ABCG: ATP binding cassette subfamily G
P450: Cytochrome P450
P4507A1: Cytochrome P450 7A1
AMPK: Adenosine 5′-monophosphate- (AMP-) activated protein kinase
HMG-CoA: 3-Hydroxy-3-methylglutaryl–coenzyme A
SREBP: Sterol regulatory element-binding protein
SHP: Small heterodimer partner
MCC: Microcrystalline cellulose
WB: Wheat bran
MAPK: Mitogen-activated protein kinase
C/EBP*α*: CCAAT/enhancer binding protein *α*
aP2: Adipocyte fatty acid-binding protein
ERK: Extracellular signal-related kinase
JNK: JUN N-terminal kinase
Soat: Sterol O-acyltransferase
ACOX: Accyl-CoA oxidase
FAS: Fatty acid synthase
FFARs: Free fatty acid receptors
GLP-1: Glucagon like peptide 1
MW: Molecular weight
PPARs: Peroxisome proliferator-activated receptors
SERPINA: Serpin family A member
FXR: Farnesol X receptor
LXR: Liver X receptor
RXR: Retinoid X receptor.
